# A transdiagnostic approach to addiction: possible targets for tDCS

**DOI:** 10.1192/j.eurpsy.2025.203

**Published:** 2025-08-26

**Authors:** M. W. Van Kernebeek

**Affiliations:** Dpt of Psychiatry and NEUR Research Group, Center for Neurosciences (C4N), Vrije Universiteit Brussel (VUB), Universitair Ziekenhuis Brussel (UZ Brussel), Brussels, Belgium

## Abstract

**Abstract:**

Transcranial Direct Current Stimulation (tDCS) has already been proven to be an effective modality in substance use disorder (SUD), often targeting the dorsolateral prefrontal cortex bilaterally. However, addiction is a very broad concept, comprising many different neurocognitive defects.

To better guide and investigate the potential benefits of tDCS for people who suffer from SUD, we shall take a transdiagnostic approach to SUD to see which other targets for tDCS might lead to additional clinical use, paving the way for personalised neurostimulation.

**Disclosure of Interest:**

None Declared

